# Mineralogy of the deep lower mantle in the presence of H_2_O

**DOI:** 10.1093/nsr/nwaa098

**Published:** 2020-05-13

**Authors:** Qingyang Hu, Jin Liu, Jiuhua Chen, Bingmin Yan, Yue Meng, Vitali B Prakapenka, Wendy L Mao, Ho-Kwang Mao

**Affiliations:** Center for High Pressure Science and Technology Advanced Research (HPSTAR), Beijing 100094, China; Center for High Pressure Science and Technology Advanced Research (HPSTAR), Beijing 100094, China; Department of Geological Sciences, Stanford University, Stanford, CA 94305, USA; Center for Study of Matter under Extreme Conditions, Department of Mechanical and Materials Engineering, Florida International University, Miami, FL 33199, USA; Center for High Pressure Science and Technology Advanced Research (HPSTAR), Beijing 100094, China; High Pressure Collaborative Access Team (HPCAT), X-ray Science Division, Argonne National Laboratory, Argonne, IL 60439, USA; Center for Advanced Radiation Sources, University of Chicago, Chicago, IL 60437, USA; Department of Geological Sciences, Stanford University, Stanford, CA 94305, USA; Center for High Pressure Science and Technology Advanced Research (HPSTAR), Beijing 100094, China

**Keywords:** lower mantle, water-mantle interaction, ferropericlase, high pressure, mantle mineralogy

## Abstract

Understanding the mineralogy of the Earth's interior is a prerequisite for unravelling the evolution and dynamics of our planet. Here, we conducted high pressure-temperature experiments mimicking the conditions of the deep lower mantle (DLM, 1800–2890 km in depth) and observed surprising mineralogical transformations in the presence of water. Ferropericlase, (Mg, Fe)O, which is the most abundant oxide mineral in Earth, reacts with H_2_O to form a previously unknown (Mg, Fe)O_2_H*_x_* (*x* ≤ 1) phase. The (Mg, Fe)O_2_H*_x_* has a pyrite structure and it coexists with the dominant silicate phases, bridgmanite and post-perovskite. Depending on Mg content and geotherm temperatures, the transformation may occur at 1800 km for (Mg_0.6_Fe_0.4_)O or beyond 2300 km for (Mg_0.7_Fe_0.3_)O. The (Mg, Fe)O_2_H*_x_* is an oxygen excess phase that stores an excessive amount of oxygen beyond the charge balance of maximum cation valences (Mg^2+^, Fe^3+^ and H^+^). This important phase has a number of far-reaching implications including extreme redox inhomogeneity, deep-oxygen reservoirs in the DLM and an internal source for modulating oxygen in the atmosphere.

## INTRODUCTION

At depths of about 660–2890 km from the Earth's surface, the lower mantle represents the largest fraction of our planet (>55% by volume). In the lower mantle, the dominant upper-mantle ferromagnesian silicate mineral (Mg, Fe)_2_SiO_4_ breaks down into (Mg, Fe)O ferropericlase (Fp) and (Mg, Fe)SiO_3_ bridgmanite (Brg) [1,2], and this main mineral assemblage further transforms to Fp and post-perovskite (pPv) near the core-mantle boundary (CMB) [3,4]. Recent experiments revealed that the lower half of the deep lower mantle (DLM) beneath 1800 km in depth may be governed by remarkably different pressure-induced chemistry, leading to a set of unexpected mineralogical, geophysical and geochemical behaviors [5–7]. In the DLM, water becomes a strong oxidant that can oxidize iron [8] and common iron oxides [9] to form hydrogen-bearing FeO_2_H*_x_* (*x* < 1) with pyrite structure (Py) [10–12]:

(1) \begin{equation*}{\rm{Fe}} + 2{{\rm{H}}_2}{\rm{O}} = {\rm{Fe}}{{\rm{O}}_2}{{\rm{H}}_x} + \left( {2 - 0.5x} \right){{\rm{H}}_2},\end{equation*}

(2) \begin{equation*}{\rm{FeO}} + {{\rm{H}}_2}{\rm{O}} = {\rm{Fe}}{{\rm{O}}_2}{{\rm{H}}_x} + \left( {1 - 0.5x} \right){{\rm{H}}_2}.\end{equation*}

However, pure iron and wüstite (FeO) are not major minerals in the lower mantle, and only become abundant when approaching the iron core; iron in the mantle is mostly present as an endmember component of ferromagnesian silicates or oxides. In the upper mantle and crust, Fe and Mg often form a complete solid solution in silicates, but in the lower mantle, they tend to be separated. Brg can only accommodate a maximum of 15 mole% Fe under the reducing DLM conditions, and the Fe-endmember silicate simply breaks down to simple oxides [13]. The key questions, therefore, are how much Mg can replace Fe to form (Mg, Fe)O_2_H*_x_* and under what *P-T* conditions? Here, we re-examined phase relations of ferromagnesian silicates and oxides in the presence of water and found very distinct mineralogy under the DLM conditions.

The polymorphic transition of olivine to wadsleyite at 410 km depth causes a seismic discontinuity that defines the top of the mantle transition zone (MTZ). With increasing depth (and pressure), wadsleyite transforms to ringwoodite and eventually decomposes to Fp plus Brg [2], causing the 660 km seismic discontinuity that defines the boundary between the MTZ and the lower mantle. Wadsleyite and ringwoodite can store 1–3 wt% water [14,15], which could make MTZ the largest water reservoir in the solid Earth. Schmandt *et al*. [16] explored the dehydration melting of hydrous peridotite at the top of the lower mantle, which may represent one of the key processes to transport water into the lower mantle [17–19]. Here, we further investigated the wadsleyite-water system down to the DLM, where dramatically different chemical behaviors lead to the creation of novel hydrous phases [20].

## RESULTS

**Figure 1.**
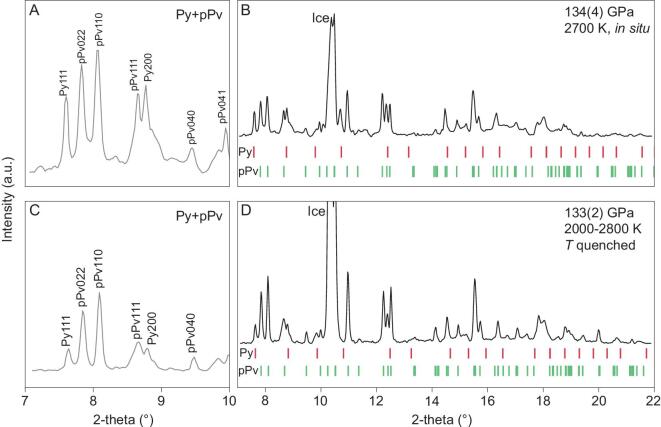
Py-phase synthesized from iron-bearing olivine (Fo80) and H_2_O. (A, B) *In situ* XRD at 2700(100) K. New sets of diffraction peaks are associated with Py-phase and post-perovskite (Mg, Fe)SiO_3_. (C, D) Quenched to RT. The X-ray wavelength was 0.3344 Å. (A, C) Zoomed in for signature high *d*-spacing peaks. Py, Py-phase; pPv, post-perovskite type (Mg, Fe)SiO_3_.

**Figure 2.**
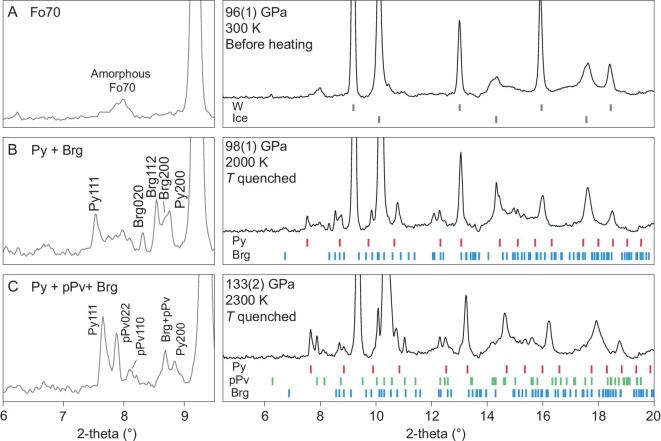
Py-phase synthesized from iron-bearing olivine (Fo70) and water. Left panels zoomed in for signature of high *d-*spacing peaks. (A) Amorphous Fo70 and H_2_O at 96 GPa before laser heating. Strong peaks are from tungsten and ice. (B) Temperature quenched from 2000 K. New sets of diffraction peaks are associated with the Py-phase and Brg. (C) Sample was compressed to 133 GPa and heated at 2300 K. The products are Py-phase and (Mg, Fe)SiO_3_ post-perovskite [3]. The X-ray wavelength was 0.3344 Å. Py, Py-phase. Brg, bridgmanite. pPv, post-perovskite type (Mg, Fe)SiO_3_.

We performed high pressure-temperature (*P-T*) X-ray diffraction (XRD) experiments using laser-heated diamond anvil cells (DACs). A synthetic wadsleyite sample with composition (Mg_0.8_Fe_0.2_)_2_SiO_4_ (Fo80) containing 2 wt% water and an olivine sample with composition (Mg_0.7_Fe_0.3_)_2_SiO_4_ (Fo70) were used as starting materials and loaded with water into the sample chambers of two DACs. Fo80 was compressed to 134 GPa and heated to 2800 K to mimic conditions of the CMB. The sample assemblage was probed *in situ* at high *P* using synchrotron XRD during laser heating (Fig. 1). Fo80 decomposed into pPv-type (Mg, Fe)SiO_3_ and a pyrite-structured oxide phase (Mg, Fe)O_2_H*_x_* (Py-phase), instead of the well-known decomposition product of Fp in the anhydrous case. The Py-phase was quenchable to room temperature (RT) at high pressure (Fig. 1C and D). The Fo70 in H_2_O was studied at 98–133 GPa, and the Py-phase was also observed to coexist with (Mg, Fe)SiO_3_ (Brg and/or pPv depending on the pressure) upon laser heating (Fig. 2). These results confirm that in the presence of H_2_O, the Py-phase would replace Fp to become an important constituent of the DLM. The dry reaction assemblage,

(3) \begin{equation*}{\left( {{\rm{Fe}},{\rm{Mg}}} \right)_2}{\rm{Si}}{{\rm{O}}_4} = {\rm{ }}\left( {{\rm{Fe}},{\rm{Mg}}} \right){\rm{Si}}{{\rm{O}}_3} + {\rm{ }}\left( {{\rm{Fe}},{\rm{Mg}}} \right){\rm{O}},\end{equation*}

is replaced by the wet mineral assemblage in the presence of water:

(4) \begin{eqnarray*}{\left( {{\rm{Fe}},{\rm{Mg}}} \right)_2}{\rm{Si}}{{\rm{O}}_4} &+& {\rm{ }}{{\rm{H}}_2}{\rm{O }} = {\rm{ }}\left( {{\rm{Fe}},{\rm{Mg}}} \right){\rm{Si}}{{\rm{O}}_3}\nonumber\\ &+& {\rm{ }}\left( {{\rm{Fe}},{\rm{Mg}}} \right){{\rm{O}}_2}{{\rm{H}}_x}\nonumber\\ &+& {\rm{ }}\left( {1 - 0.5{\rm{x}}} \right){{\rm{H}}_2}.\end{eqnarray*}

We proceeded to investigate the *P-T* dependence of the Mg fraction *X_Mg_* = Mg/(Fe+Mg), and hydrogen content *x* in the wet assemblage. We note that the water in Eq. (4) mainly affects the silica-free oxides. The study can thus be simplified to the silica-free Fe-Mg-O-H system of the Fp to Py-phase conversion by substituting Eq. (3) into Eq. (4):

(5) \begin{eqnarray*} \left( {{\rm{Fe}},{\rm{Mg}}} \right){\rm{O }}\left( {{\rm{Fp}}} \right) &+& {{\rm{H}}_2}{\rm{O}} = \left( {{\rm{Fe}},{\rm{Mg}}} \right){{\rm{O}}_2}{{\rm{H}}_x}\left( {{\rm{Py}}} \right)\nonumber\\ &+& \left( {1 - 0.5{\rm{x}}} \right){{\rm{H}}_2}. \end{eqnarray*}

**Figure 3.**
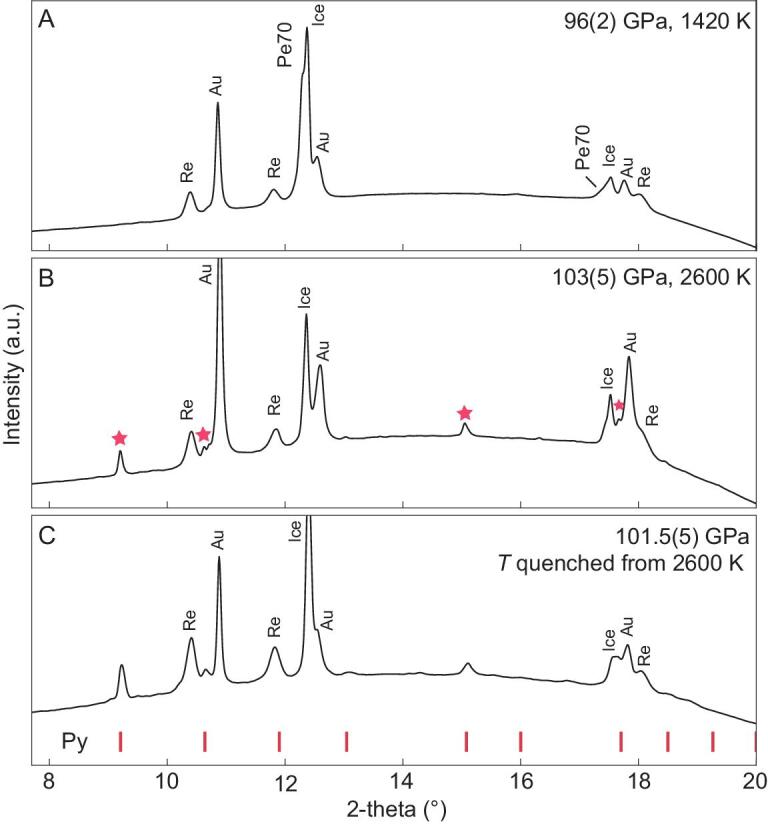
Py-phase synthesized from Pe70 and H_2_O. (A) *In situ* X-ray diffraction pattern of Pe70+H_2_O at 96 GPa and 1420 K. (B) Same as (A) but at 103 GPa and 2600 K. Peaks labeled by stars are from the Py-phase. (C) Same as (A) but at 101.5 GPa after quenching to RT. Red hash marks indicate peaks from the Py-phase. The X-ray wavelength was 0.4066 Å. We estimate ±200 K error in temperature. Py, Py-phase.

**Figure 4.**
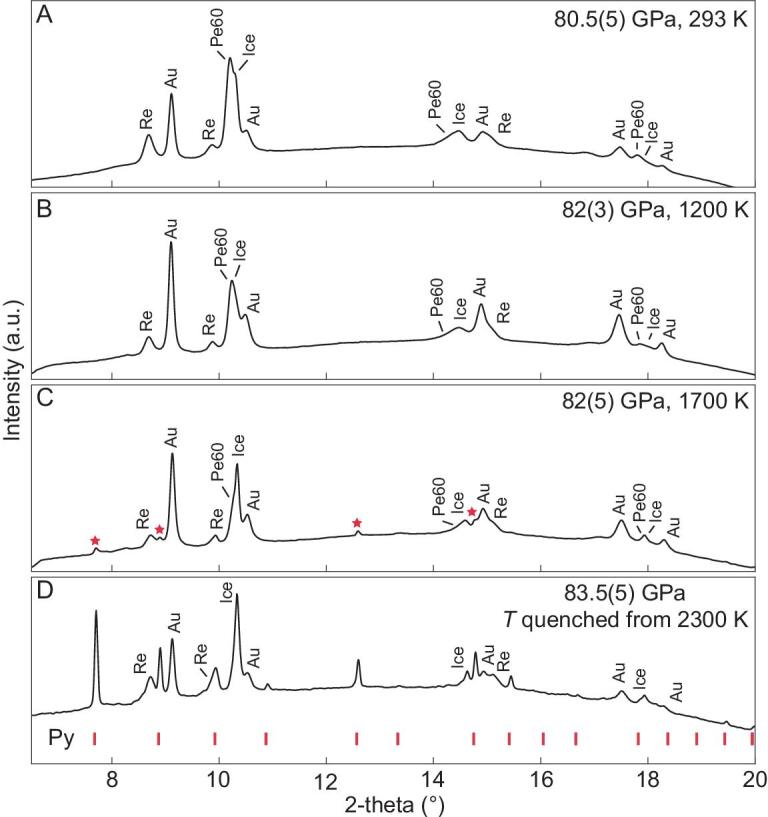
Py-phase synthesized from Pe60 in H_2_O. *In situ* X-ray diffraction patterns were collected at the center of laser heating spot. Pe60+H_2_O at 80.5 GPa and ambient temperature (A), at 82 GPa and 1200 K (B), and at 82 GPa and 1700 K (C). Stars indicate peaks from the Py-phase. (D) Same as (A) but at 83.5 GPa after quenching back to ambient temperature with red hash marks noting the Py-phase. Uncertainties in pressure were estimated from measurement error and the thermal expansion of high *P-T* gold [41]. The X-ray wavelength was 0.3445 Å. Temperature uncertainty is ±200 K in this experiment. Py, Py-phase.

Thus, we studied the effects of water on the high-pressure chemistry of Fp. As the most abundant oxide in the mantle, Fp has been extensively investigated for its elastic, optical, thermal and elemental partitioning properties to understand the geophysical and geochemical behavior of the lower mantle (e.g. [21,22]). As a result of the very strong partitioning of Mg in Brg relative to Fp, we chose three starting Fp compositions with *X_Mg_* = 80% (Pe80), 70% (Pe70) and 60% (Pe60), which would be in equilibrium with Brg of *X_Mg_* = 96%, 92% and 88% [23,24], respectively, in representative pyrolite compositions with bulk *X_Mg_* of 90–80%. The Pe70 sample was compressed in H_2_O to 96 GPa and laser-heated to 1460 K. Its XRD patterns clearly show coexistence of body-centered cubic ice-VII [17] and face-centered cubic Fp (Fig. 3A). Above 103 GPa and 1800 K, Eq. (5) proceeds to the right and the Fp-(Mg_0.7_Fe_0.3_)O sample reacted with H_2_O to form Py-(Mg_0.7_Fe_0.3_)O_2_H*_x_* (Fig. 3B). The Py-phase is quenchable to RT under pressure (Fig. 3C). Releasing pressure at RT, the Py-phase was preserved metastable down to 50 GPa, and is unstable at lower pressures (Supplementary Figs S1 and S2). A similar transition to the Py-phase was observed with Pe60 as the starting material (Fig. 4), but the transition pressure was ∼20 GPa lower than that of Pe70. The transition was not observed in the experiment with Pe80 up to 110 GPa and 2450 K, where Pe80 remained in the ferropericlase phase without the Py-phase.

We performed conclusive crystallographic analysis on the Py-phase using a multigrain algorithm [25,26]. The algorithm picked up 10 single-crystal grains from the quenched Py-(Mg_0.6_Fe_0.4_)O_2_H*_x_* sample after laser heating at 83.5 GPa and unambiguously indexed each single crystal to the Py-phase cubic lattice of the $Pa\bar{3}$ space group with its own individual orientation matrix. The crystallographic data are presented in Supplementary Tables S1 and S2. The redundant measurements yield accurate determination of lattice parameter, *a* = 4.4403(16) Å, and volume per formula unit (f.u.), *V* = 21.877(24) Å^3^, for the Py-(Mg_0.6_Fe_0.4_)O_2_H*_x_* at 83.5 GPa and RT.

**Figure 5.**
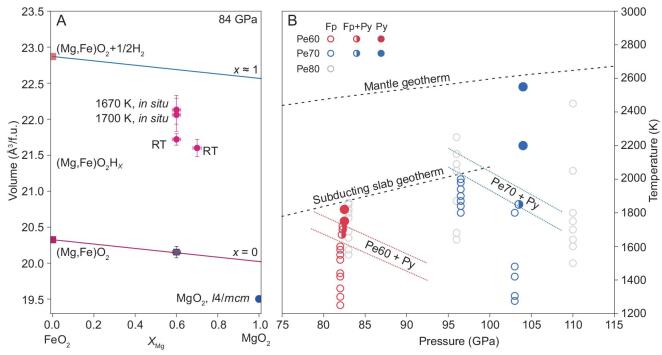
*P-T* phase relations for ferropericlase with H_2_O. (A) Projected *V*-*X*_Mg_ relation at 84 GPa. The Py-MgO_2_ endmember is 2.6% higher in volume than the stable *I*4*/mcm* phase (the blue dot) [44]. Volume of (Mg_0.7_, Fe_0.3_)O was decompressed from higher pressure to 84 GPa. f.u., formula unit. (B) Open and filled circles represent the observations of only Fp phase or the Py-phase, respectively. Half-filled circles indicate the coexistence of Fp and Py-phase. Volumes of phase-I hydrogen are from Ref. [45]. Pressure has uncertainty up to ±6 GPa at 104 GPa and 2550 K (see Methods). The uncertainty in laser heating temperature is ±200 K.

The unit-cell volume per f.u. of Py-(Mg, Fe)O_2_ is a function of *P-T*-*X_Mg_-x* (Fig. 5A). To identify the effects of different variables, we conducted an additional H-free high *P-T* experiment of (Mg_0.6_Fe_0.4_)O sample loaded in pure O_2_ medium without any water. We successfully synthesized H-free Py-(Mg_0.6_Fe_0.4_)O_2_ at 84 GPa, and after quenching to RT, determined its volume per f.u. to be 20.127(6) Å^3^ (Fig. 5A). Based on the reported volume [10] of Mg-free Py-FeO_2_ as 20.304 Å^3^, the volume dependence of H-free Py-(Mg, Fe)O_2_ on *X_Mg_* at 84 GPa and RT is represented by the linear relationship:

(6) \begin{equation*}{V_{Py}}\left( {{{\mathring{\rm A}}^3}} \right) = 20.304{\rm{ }}\left( {1-0.015{X_{Mg}}} \right),\end{equation*}

where 0 < *X_Mg_* < 1. The hydrogen content *x* can be estimated by comparing the molar volumes of hydrogen-bearing and hydrogen-free Py-phases of the same *X_Mg_*. Following the scheme [9] used for FeO_2_H*_x_*, hydrogen content *x* in (Mg, Fe)O_2_H*_x_* synthesized at 83.5 GPa is estimated to be between 0.6 and 0.8 (Fig. 5A).

We further examined the cation composition of the recovered sample after complete decompression to ambient pressure. A recovered Py-(Mg_0.6_Fe_0.4_)O_2_H*_x_* sample was analyzed using an electron microprobe (Supplementary Fig. S3). Although the sample possibly lost hydrogen and excess oxygen at ambient conditions, the Mg to (Mg+Fe) ratio (*X_Mg_*) remained uniform at 0.59 ± 0.04, indicating preservation of the cation composition during the conversion of Fp to Py-(Mg, Fe)O_2_H*_x_*.

## IMPLICATIONS

Our high *P-T* experiments on the phase relations of Pe60, Pe70 and Pe80 are summarized in Fig. 5B, a projection of Fe-Mg-O-H quaternary phase diagram. The results clearly demonstrate that extensive solid solutions can be formed in Py-(Fe,Mg)O_2_H*_x_*, and the maximum Mg limit increases with increasing *P*. The results indicate that for a wet pyrolite with an average bulk composition of *X_Mg_* = 85%, its Fp (Pe70) will transform to the Py-phase at depths of 2100–2300 km depending upon the hot or cold geotherm temperatures [27]. For an iron-rich bulk pyrolite *X_Mg_* = 80%, its Fp (Pe60) will transform to the Py-phase at 1700–1900 km depth, while for an iron-poor bulk *X_Mg_* = 90%, its Fp (Pe80) may remain stable down to the CMB at 2891 km depth. Nevertheless, with essentially inexhaustible iron from the core, reaction (1) will kick in at the CMB to provide sufficient FeO_2_H*_x_* endmember to stabilize (Fe,Mg)O_2_H*_x_.*

**Figure 6.**
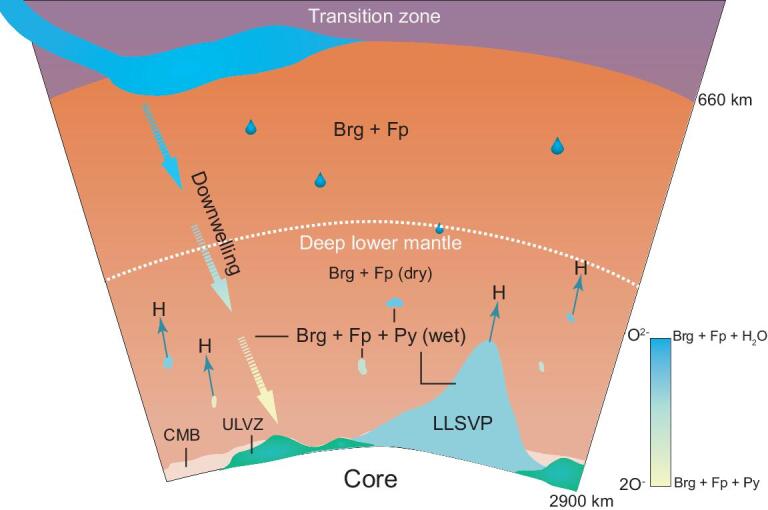
Mineralogy at the lower mantle. The lower mantle is divided roughly in the middle of the lower mantle, below which is the DLM. While H_2_O or dense hydrous phases (blue droplets) are carried down by plate subduction or form from primitive water in shallower lower mantle, they react with Fp to form the Py-phases in the DLM (solid patches). Color gradient in the patches indicates the content of Py-phases. The mineral composition within the wet pockets of the DLM may include Brg, Fp as well as Py-(Mg, Fe)O_2_H*_x_*.

As a result of the ubiquitous presence of Fp in the lower mantle, its transition to the Py-(Mg, Fe)O_2_H*_x_* carries great geological significance. Our experiments suggest that the mineralogical heterogeneity of the deep lower mantle depends upon wet or dry conditions (Fig. 6). How much water (carried by hydrous materials or as free solid or liquid H_2_O) is available in the DLM remains an open question [8,28]. This relies heavily on the primordial water reservoirs and distributions [29] in the DLM as well as water circulation from the MTZ [19,30]. Previous views of a dry lower mantle were largely caused by a lack of hydrous phases that could carry water under high *P-T* conditions beyond the MTZ. Since the discoveries of the dense hydrous phase-H [20] and δ-AlOOH [31], hydration of the lower mantle has become an increasingly more likely scenario, which has been further strengthened by a series of subsequent observations of high *P-T* hydrogen-bearing phases [11,32–34]. In addition, the breakdown of water-bearing wadsleyite and ringwoodite to anhydrous Brg and Fp does not prohibit the descent of water; the released H_2_O could be carried down as inclusions in minerals [17], trapped between grain boundaries or transferred to the newly discovered high *P-T* hydrogen-bearing phases [35].

The remarkable properties and geological implications of the iron endmember Py-FeO_2_H*_x_* have been studied extensively [5,11,32,36], and can be used as guidance for the Py-(Mg, Fe)O_2_H*_x_* solid solutions. The hydrogen released from the reaction (3) in the DLM can rise freely to sustain the water–hydrogen cycle [9]. The observed low seismic velocities of Py-FeO_2_H*_x_* have been used to interpret the seismologically observed ultralow velocity zones (ULVZs) [36]. In fact, the velocities of the iron endmember are too low and some other mantle materials must be added to match the ULVZ. Likewise, Py-(Mg, Fe)O_2_H*_x_* is expected to have low velocities, but not as low as that of the iron endmember because of the presence of Mg, thus becoming a better fit for matching ULVZs.

## CONCLUSION

Both Py-(Mg, Fe)O_2_H*_x_* and Py-FeO_2_H*_x_* are nominally oxygen excess phases, which are defined as the total oxygen exceeding the balance of the cations based on the ‘normal’ maximum valence state assignment of Mg^2+^, Fe^3+^, H^+^ and O^2−^. These phases could create highly inhomogeneous and unusual redox environments in the DLM [37], collect into oxygen-rich reservoirs and potentially provide a huge internal source of oxygen [10] for modulating oxygen fluctuations in the atmosphere [38,39]. Our experiments indicate that the Py-type phases cannot retain all excess oxygen after releasing pressure, thus preventing direct recovery from natural samples. They may convert to low-pressure phases with the maximum valence state, and reveal the oxidation history. This provides an alternative explanation for the high ferric inclusions often observed in natural diamonds [37,40]. In conclusion, the presence of water in DLM will create a very different mineralogy enriched in Py-(Mg, Fe)O_2_H*_x_* that has a profound impact on the evolution and dynamics of the solid Earth.

## METHODS

### Sample synthesis and characterization

#### Hydrous wadsleyite

Hydrous wadsleyite samples were synthesized with a Kawai-type 1500-ton multi-anvil apparatus at the Center for High Pressure Science and Technology Advanced Research (HPSTAR). The reaction precursors were mixed in the optimized molar ratio (0.4 FeO:1.6 Mg(OH)_2_:1.0 SiO_2_) and then placed in an alloy capsule made of 75% Au and 25% Pd. The Fo80 samples were synthesized at 16 GPa and heated at 1400°C for 4 hours. After samples were quenched and decompressed, the recovered samples were checked on the synchrotron beamline by XRD to be single phase wadsleyite (Fig. 1). The water content was measured from infrared-red spectroscopy and determined to be 2.2±0.3 wt% (details in Supplementary Fig. S4).

#### Olivine (Fo70)

Fo70 sample was synthesized at HPSTAR. The starting materials consisted of reagent grade MgO, Fe_2_O_3_ and SiO_2_ in the proper ratio. They were loaded into separate crucibles and placed in an oven at 1600°C for more than 8 hours. After mixing well, synthesis was performed in a gas-mixing furnace for >2 hours at 1300°C and oxygen fugacity buffer of Fayalite-Magnetite-Quartz -1.

#### Ferropericlase (Mg_0.7_Fe_0.3_)O and (Mg_0.6_Fe_0.4_)O

We synthesized ferropericlase and olivine samples using the piston-cylinder apparatus. Fp compositions were synthesized at the Geophysical Laboratory, Carnegie Institution of Washington. MgO and Fe_2_O_3_ powders were mixed according to the target mole ratio of Mg and Fe. The mixture was placed in a platinum crucible and kept in a furnace at 1300°C for 24 hours for dehydration. A solid solution of cubic (Mg, Fe)O was synthesized from the mixture of oxides at 1 GPa and 1200°C in a piston-cylinder press using a graphite capsule, which reduced ferric to ferrous iron. The composition and homogeneity of the synthesized samples were confirmed by electron probe microanalysis (Supplementary Fig. S5).

#### *In situ* XRD experiments at high *P-T* conditions

Angular dispersive XRD experiments were performed at sectors 16ID-B of HPCAT and 13ID-D of GSECARS at the APS, ANL. The x-ray energy was 30.5 keV for the samples of Pe70 in H_2_O, Pe60 in O_2_, 36.0 keV for Pe60 in water and 37.0 keV for Fo70 in water. Samples were pre-compressed in a pair of 1 mm diamond anvils and sample platelets of ∼30 (W) × 30 (L) × 10 (T) μm^3^ were loaded into DACs. High pressure was achieved using beveled diamond anvils with 300 μm outer diameter and 150 or 100 μm inner diameter culet. The sample chamber was a hole of one-third of the inner culet diameter, which was drilled in a tungsten or rhenium gasket. For the experiments with H_2_O, a droplet of deionized water was injected into the chamber and then was immediately sealed. For Fp+O_2_ experiment, liquefied O_2_ was piped into the sample chamber in a liquid-nitrogen cooled container. One to two pieces of thin gold foil were placed around the sample as pressure calibrant. Combining the uncertainties from indexing (±0.3 GPa) and calibrant (±1.0 GPa), the total pressure uncertainties propagated to ±1.1 GPa. During *in situ* laser heating, thermal pressure was corrected for thermal effects using a *P-V-T* equation of states of gold [41] and uncertainty was up to ±6 GPa.

The diameter of laser spot at GSECARS was around 20 μm and flat down to ∼10 μm. At the time of experiment, the incident X-ray beam size was 3 × 4 μm^2^. We estimated temperature uncertainties during laser heating were up to ±200 K.

At HPCAT, the heating temperature was measured by fitting the gray-body radiation curve on both sides of the sample. The diameter of laser spot was approximately 30 μm at 2000 K, which covered the entire sample. We estimated an uncertainty of up to ±200 K on temperature measurements throughout the laser heating experiments [42,43].

## Supplementary Material

nwaa098_Supplemental_File
