# Supplementary material for: Mineralogy of the deep lower mantle in the presence of H2O
Source: Natl Sci Rev. 2020 May 13;8(4):nwaa098. doi: 10.1093/nsr/nwaa098 (PMC8288427; doi:10.1093/nsr/nwaa098)
Supplement: nwaa098_Supplemental_File [file nwaa098_supplemental_file.docx]

**Supplementary Data**

**Mineralogy of the deep lower mantle in the presence of H_2_O**

Qingyang Hu^1^, Jin Liu^1,2^*, Jiuhua Chen^3^, Bingmin Yan^1^, Yue Meng^4^, Vitali B. Prakapenka^5^, Wendy L. Mao^2^ and Ho-kwang Mao^1^*

**Affiliations:**

^1^Center for High Pressure Science and Technology Advanced Research (HPSTAR), Beijing 100094, PRC

^2^Department of Geological Sciences, Stanford University, Stanford, CA 94305, USA

^3^Center for Study of Matter under Extreme Conditions, Department of Mechanical and Materials Engineering, Florida International University, Miami, FL 33199, USA

^4^High Pressure Collaborative Access Team (HPCAT), X-ray Science Division, Argonne National Laboratory, Argonne, IL 60439, USA

^5^Center for Advanced Radiation Sources, University of Chicago, Chicago, IL 60437, USA


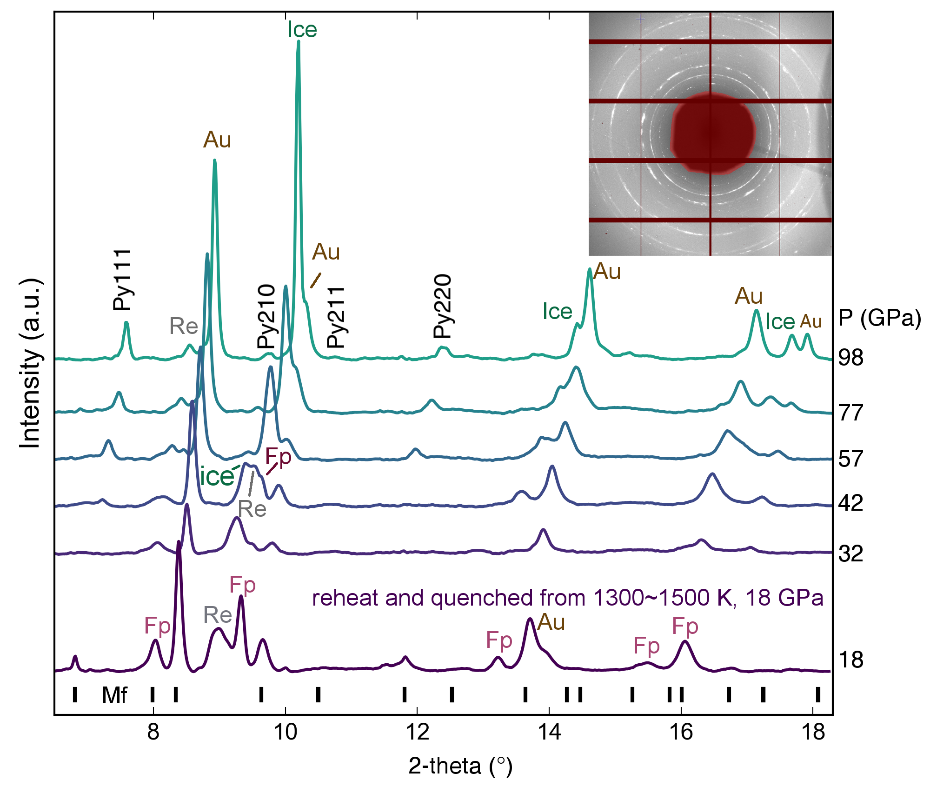


**Supplementary Fig. S1. XRD patterns for the (Mg_0.7_Fe_0.3_)O_2_H*_x_* upon decompression.** Py-phase were observable as low as 42 GPa. It completely disappeared when pressure was released to 32 GPa. Sample was heated at 18 GPa to 1300~1500 K and a new set of peaks from spinel-MgFe_2_O_4_ magnesioferrite appeared. The major diffraction peaks from the reheated sample were indexed to Mf, Fp and water. While previous experiments indicated under such *P-T* condition, Mf may decompose to Mg_2_Fe_2_O_5_ and hematite [[1](#_ENREF_1), [2](#_ENREF_2)] or further transform to a post-perovskite polymorph (hp-MgFe_2_O_4_)[[3](#_ENREF_3)], they were not observed in our experiment. Although either Mg_2_Fe_2_O_5_ or hp-MgFe_2_O_4_ may co-exist in the reheated sample, the remaining diffraction peaks are insufficient to index any of those phases. The following reaction depicts the decomposition of Py-(Mg,Fe)O_2_H*_x_* (also from EPMA data in Supplementary Fig. S5):

(Mg,Fe)O_2_H*_x_* = (Mg, Fe)Fe_2_O_4_ + 2(Mg,Fe)O + 2*x*H_2_O + (1-*x*)O_2_

At 18 GPa, the lattice parameter for α-MgFe_2_O_4_ is = 7.964(5) Å. Inset: 2-dimensional XRD pattern at 98 GPa. The incident x-ray wavelength was 0.3344 Å. Abbreviation: Mf, magnesioferrite MgFe_2_O_4_.


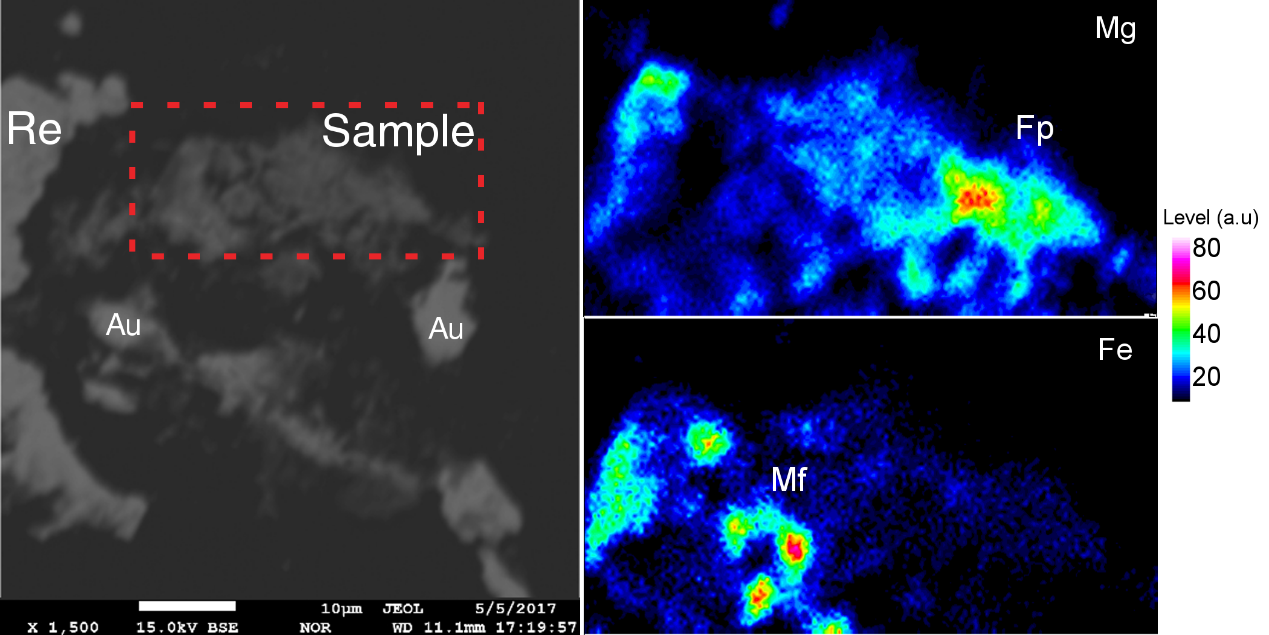


**Supplementary Fig. S2. Results from electron probe microanalysis (EPMA) for quenched (Mg_0.7_Fe_0.3_)O_2_H*_x_* sample.** Sample was reheated at to 1300~1500 K at 18 GPa. Measurements were taken at ambient pressure and RT. From mapping of Mg/Fe ratio, the sample decomposed into Fe concentrated MgFe_2_O_4_ (magnesioferrite: Mf) and Mg-rich Fp.


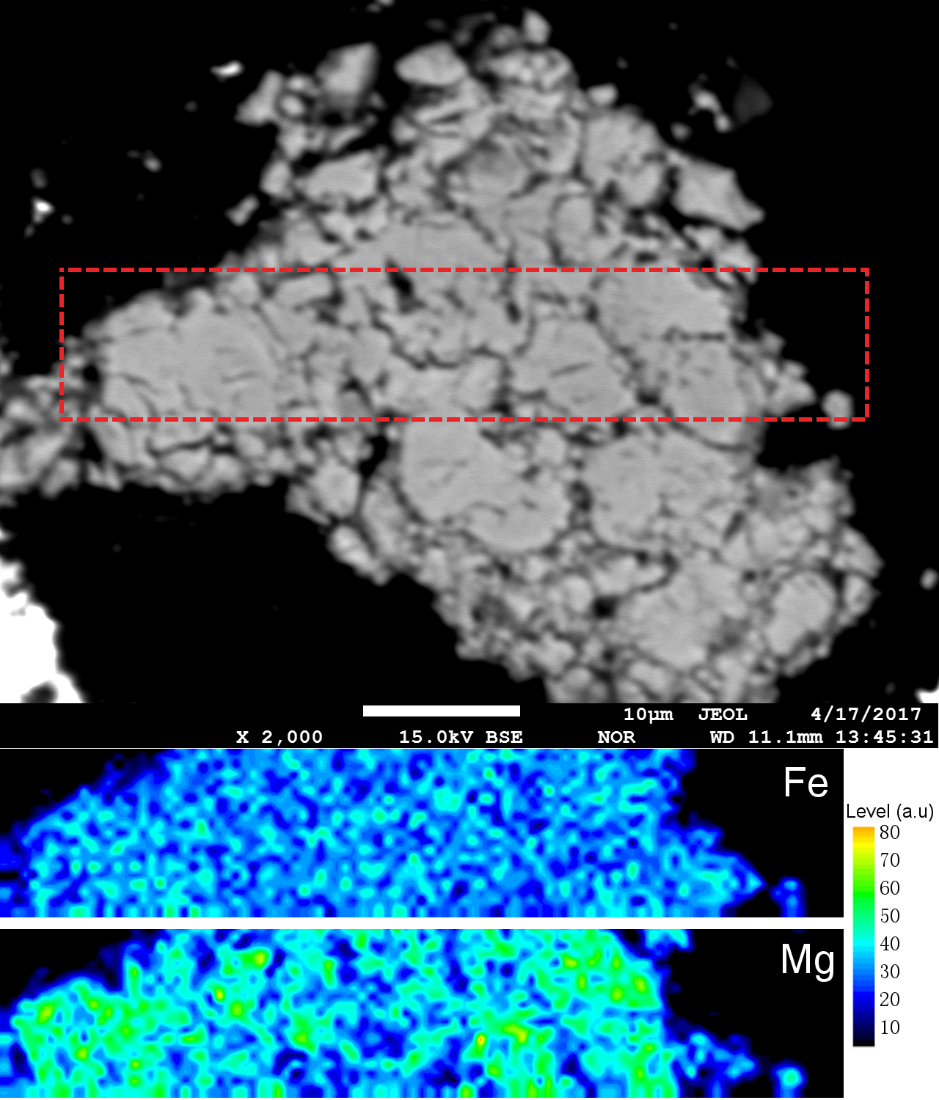


**Supplementary Fig. S3. Results from EPMA for quenched (Mg_0.6_Fe_0.4_)O_2_ sample.** Sample was quenched from 84 GPa and 2400 K and cold decompressed to ambient pressure. Due to the tiny amount of sample, it was not polished. Variation of element levels may reflect the roughness of sample surface. In the lower panels (red box region in the upper panel), the region averaged Mg/Fe ratio is 58.6(3)/41.4(2) which is consistent with the original Pe60 composition.


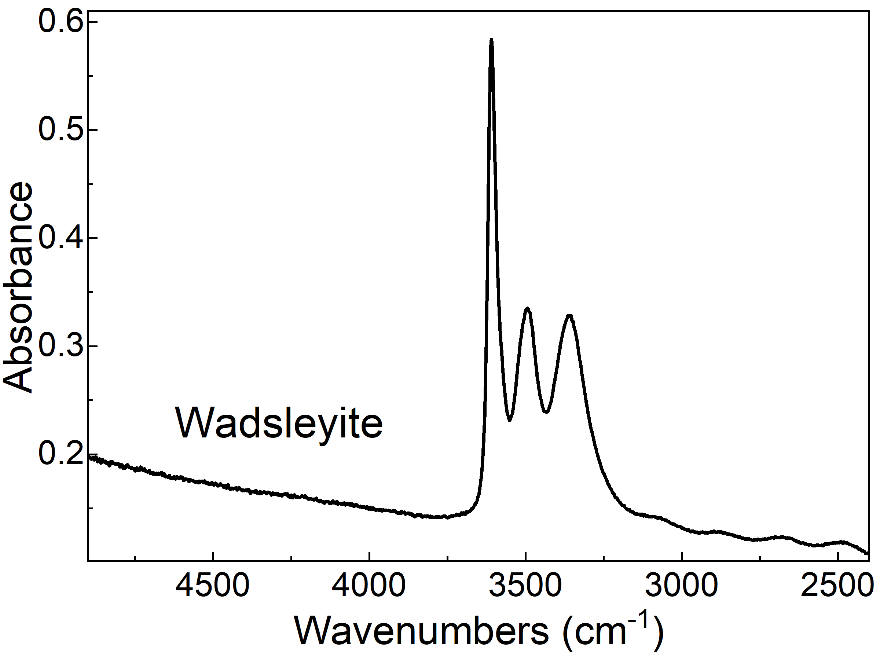


**Supplementary Fig. S4. Representative infrared-red spectra of wadsleyite sample at ambient conditions.** The hydrous wadsleyite samples were synthesized under the water-saturated conditions. The water content of 2.2±0.3 wt% was determined by a linear calibration curve by Libowitzky and Rossman [[4](#_ENREF_4)].


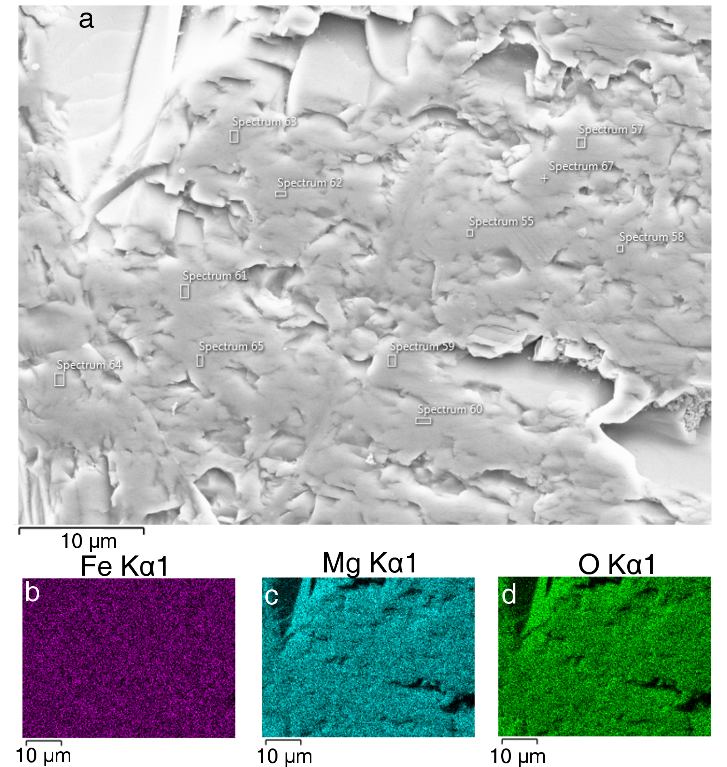


**Supplementary Fig. S5. SEM-Energy Dispersive Spectroscopy (EDS) analysis of the starting Pe70 sample. a,** An SEM image. The composition of (Mg_0.71(2)_Fe_0.29(2)_)O was averaged from 10 specimen. **b,** Fe *K* shell, **c,** Mg *K* shell and **d,** O *K* shell imaging maps.

**Supplementary Table S1.** Lattice parameters for ten grains of the Py-phase (Mg_0.6_Fe_0.4_)O_2_H*_x_* at 83.5(5) GPa. The indexing uncertainty for each grain is calculated by a non-linear least square fitting algorithm. Volume differences (*V*_diff_) per formula unit (f.u.) are compared to the average volume. The maximum-minimum volume difference is 0.36%.

| **Grain** | ***a*(Å)** | ***σ_a_*(Å)** | ***V*(Å*^3^/*f.u*.*)** | ***σ_V_*(Å^3^/f.u.)** | ***V*_diff._** |
| --- | --- | --- | --- | --- | --- |
| 1 | 4.4402 | 0.0007 | 21.885 | 0.010 | -0.01% |
| 2 | 4.4404 | 0.0007 | 21.888 | 0.010 | 0.01% |
| 3 | 4.4420 | 0.0008 | 21.912 | 0.012 | 0.12% |
| 4 | 4.4379 | 0.0006 | 21.851 | 0.009 | -0.16% |
| 5 | 4.4406 | 0.0006 | 21.891 | 0.009 | 0.02% |
| 6 | 4.4410 | 0.0008 | 21.897 | 0.012 | 0.05% |
| 7 | 4.4432 | 0.0008 | 21.929 | 0.012 | 0.20% |
| 8 | 4.4397 | 0.0007 | 21.878 | 0.010 | -0.04% |
| 9 | 4.4401 | 0.0008 | 21.884 | 0.012 | -0.01% |
| 10 | 4.4378 | 0.0007 | 21.850 | 0.010 | -0.17% |
| average | 4.4403 | 0.0016 | 21.877 | 0.024 |  |

**Supplementary Table S2.** Selected multi-grain XRD data for five crystals in Supplementary Table S1. **a** Single crystal XRD peaks for a selected Py-phase crystalite with unit-cell parameter *a* = 4.4402(7) Å (space group $Pa\bar{3}$, incident beam energy 30.5 *k*eV). The Bragg angle 2*θ*, rotation angle *ω* and azimuthal angle *η* are calculated from the orientation matrix. **b-e**, XRD indexing for the second, third, fourth and fifth Py-phase crystalite, with unit-cell parameters *a* = 4.4404(7) Å, 4.4406(6) Å, *a* = 4.4410(8) Å and *a* = 4.4397(7) Å, respectively.

**a**

| ***h*** | ***k*** | ***l*** | **2 (°)** | **  (°)** | ** ** | ***d_obs_* (Å)** | ***d_cal_* (Å)** | **Δ*d/d*** |
| --- | --- | --- | --- | --- | --- | --- | --- | --- |
| 0 | 0 | 2 | 8.89 | -12.97 | 168.39 | 2.223 | 2.220 | -0.11% |
| -1 | -1 | -2 | 10.90 | 5.50 | 312.63 | 1.814 | 1.813 | -0.05% |
| -1 | -1 | 2 | 10.90 | 3.36 | 202.60 | 1.814 | 1.813 | -0.05% |
| 1 | 1 | 2 | 10.90 | -9.50 | 132.72 | 1.814 | 1.813 | -0.05% |
| -2 | -2 | 0 | 12.60 | 1.33 | 257.70 | 1.570 | 1.570 | 0.01% |
| 2 | 2 | 0 | 12.60 | -11.68 | 77.81 | 1.570 | 1.570 | 0.01% |
| -1 | -1 | -3 | 14.77 | 11.10 | 322.94 | 1.340 | 1.339 | -0.10% |
| -1 | -1 | 3 | 14.79 | 15.70 | 191.06 | 1.338 | 1.339 | 0.04% |
| 1 | 1 | 3 | 14.78 | -13.20 | 143.16 | 1.339 | 1.339 | -0.03% |
| -2 | -2 | -2 | 15.45 | 4.88 | 293.14 | 1.281 | 1.282 | 0.03% |
| -2 | -2 | 2 | 15.45 | 3.94 | 222.02 | 1.281 | 1.282 | 0.03% |
| 2 | 2 | -2 | 15.45 | -19.08 | 42.23 | 1.281 | 1.282 | 0.03% |
| 2 | 2 | 2 | 15.44 | -11.85 | 113.30 | 1.282 | 1.282 | -0.04% |
| 1 | 2 | -3 | 16.67 | -4.00 | 23.01 | 1.188 | 1.187 | -0.13% |
| -3 | -3 | -1 | 19.48 | 5.50 | 270.99 | 1.018 | 1.019 | 0.05% |
| 3 | 3 | 1 | 19.48 | -14.33 | 91.16 | 1.018 | 1.019 | 0.05% |
| -3 | -3 | 1 | 19.48 | 5.00 | 244.11 | 1.018 | 1.019 | 0.05% |
| 3 | 3 | -1 | 19.48 | -16.63 | 64.32 | 1.018 | 1.019 | 0.05% |

**b**

| ***h*** | ***k*** | ***l*** | **2 (°)** | **  (°)** | ** ** | ***d_obs_* (Å)** | ***d_cal_* (Å)** | **Δ*d/d*** |
| --- | --- | --- | --- | --- | --- | --- | --- | --- |
| -2 | 0 | 0 | 8.90 | -8.7 | 210.10 | 2.220 | 2.220 | 0.01% |
| 0 | 0 | -2 | 8.90 | -2.5 | 300.01 | 2.220 | 2.220 | 0.01% |
| 0 | 0 | 2 | 8.90 | -12.7 | 120.13 | 2.220 | 2.220 | 0.01% |
| -1 | 0 | 2 | 9.95 | -12.6 | 146.79 | 1.986 | 1.986 | -0.02% |
| 1 | 0 | -2 | 9.95 | 5.4 | 326.62 | 1.986 | 1.986 | -0.02% |
| 1 | 0 | 2 | 9.95 | -14.9 | 93.59 | 1.986 | 1.986 | -0.02% |
| -2 | 0 | -2 | 12.60 | -4.5 | 255.00 | 1.570 | 1.570 | 0.01% |
| -2 | 0 | 2 | 12.59 | -18.3 | 165.88 | 1.571 | 1.570 | -0.06% |
| 2 | 0 | 2 | 12.60 | -17.7 | 75.18 | 1.570 | 1.570 | 0.01% |
| -3 | -1 | -1 | 14.78 | 19.5 | 228.63 | 1.339 | 1.339 | -0.03% |
| -1 | -1 | -3 | 14.78 | 16.0 | 279.61 | 1.339 | 1.339 | -0.03% |
| 1 | 1 | 3 | 14.78 | 1.0 | 99.81 | 1.339 | 1.339 | -0.03% |
| 3 | 1 | 1 | 14.77 | -0.3 | 48.86 | 1.340 | 1.339 | -0.09% |
| 0 | 0 | -4 | 17.85 | 2.4 | 300.24 | 1.110 | 1.110 | -0.02% |
| 0 | 0 | 4 | 17.85 | -17.8 | 120.47 | 1.110 | 1.110 | -0.02% |
| -3 | -1 | -3 | 19.46 | 12.5 | 254.13 | 1.019 | 1.019 | -0.05% |
| 3 | 1 | 3 | 19.47 | -7.7 | 74.32 | 1.019 | 1.019 | 0.00% |
| -4 | 0 | -2 | 20.00 | -1.1 | 236.12 | 0.992 | 0.993 | 0.10% |
| -2 | 0 | -4 | 19.99 | 0.1 | 273.53 | 0.992 | 0.993 | 0.05% |
| 2 | 0 | -4 | 19.97 | 14.5 | 327.52 | 0.993 | 0.993 | -0.05% |
| 2 | 0 | 4 | 20.00 | -20.0 | 93.72 | 0.992 | 0.993 | 0.10% |

**c**

| ***h*** | ***k*** | ***l*** | **2q (°)** | **  (°)** | ** ** | ***d_obs_* (Å)** | ***d_cal_* (Å)** | **Δ*d/d*** |
| --- | --- | --- | --- | --- | --- | --- | --- | --- |
| 0 | 0 | -2 | 8.90 | -4.4 | 197.01 | 2.220 | 2.220 | 0.01% |
| -1 | -2 | 1 | 10.90 | -10.5 | 131.41 | 1.814 | 1.813 | -0.04% |
| -1 | -1 | 2 | 10.90 | -3.2 | 50.74 | 1.814 | 1.813 | -0.04% |
| 1 | 2 | -1 | 10.90 | -1.5 | 262.97 | 1.814 | 1.813 | -0.04% |
| 1 | 2 | 1 | 10.90 | 3.9 | 311.21 | 1.814 | 1.813 | -0.04% |
| -1 | -1 | 3 | 14.80 | -10.5 | 40.70 | 1.337 | 1.339 | 0.11% |
| 1 | 1 | -3 | 14.79 | 12.1 | 220.46 | 1.338 | 1.339 | 0.04% |
| 1 | 3 | -1 | 14.79 | -6.8 | 269.96 | 1.338 | 1.339 | 0.04% |
| 1 | 3 | 1 | 14.80 | -4.4 | 305.32 | 1.337 | 1.339 | 0.11% |
| -2 | -2 | 2 | 15.43 | -0.5 | 69.92 | 1.283 | 1.282 | -0.10% |
| 2 | 2 | -2 | 15.44 | 16.2 | 249.75 | 1.282 | 1.282 | -0.03% |
| -2 | -3 | 0 | 16.08 | -6.4 | 106.61 | 1.232 | 1.232 | 0.00% |
| -2 | -3 | -1 | 16.69 | -5.9 | 122.23 | 1.187 | 1.187 | 0.00% |
| -2 | -3 | 1 | 16.69 | -8.3 | 91.08 | 1.187 | 1.187 | 0.00% |
| 2 | 3 | -1 | 16.68 | 8.5 | 270.90 | 1.188 | 1.187 | -0.06% |
| -1 | -2 | 3 | 16.69 | -20.0 | 53.64 | 1.187 | 1.187 | 0.00% |
| 1 | 2 | -3 | 16.71 | 1.2 | 233.42 | 1.185 | 1.187 | 0.11% |
| 0 | 0 | -4 | 17.85 | 11.9 | 195.04 | 1.110 | 1.110 | -0.01% |
| -1 | -1 | -4 | 18.94 | 5.7 | 176.44 | 1.047 | 1.047 | -0.02% |
| 1 | 3 | 3 | 19.46 | 8.4 | 332.35 | 1.019 | 1.019 | -0.05% |
| -2 | -4 | 0 | 19.98 | -16.2 | 107.45 | 0.993 | 0.993 | 0.00% |
| 2 | 4 | 0 | 19.98 | 4.7 | 287.25 | 0.993 | 0.993 | 0.00% |

**d**

| ***h*** | ***k*** | ***l*** | **2 (°)** | **  (°)** | ** ** | ***d_obs_* (Å)** | ***d_cal_* (Å)** | **Δ*d/d*** |
| --- | --- | --- | --- | --- | --- | --- | --- | --- |
| -1 | 1 | 2 | 10.90 | -13.5 | 82.34 | 1.814 | 1.813 | -0.03% |
| 1 | -1 | -2 | 10.90 | -2.5 | 262.22 | 1.814 | 1.813 | -0.03% |
| 1 | 2 | -1 | 10.90 | 4.3 | 343.29 | 1.814 | 1.813 | -0.03% |
| 2 | 0 | -2 | 12.59 | 12.5 | 288.97 | 1.571 | 1.570 | -0.05% |
| -1 | 3 | 1 | 14.78 | 4.6 | 44.20 | 1.339 | 1.339 | -0.01% |
| -2 | -2 | 2 | 15.44 | -14.3 | 144.66 | 1.282 | 1.282 | -0.02% |
| -2 | 2 | 2 | 15.45 | 2.2 | 73.81 | 1.281 | 1.282 | 0.04% |
| 2 | 2 | -2 | 15.45 | 12.0 | 324.40 | 1.281 | 1.282 | 0.04% |
| -2 | -1 | 3 | 16.69 | -19.5 | 122.96 | 1.187 | 1.187 | 0.00% |
| 2 | 1 | -3 | 16.69 | 0.5 | 302.72 | 1.187 | 1.187 | 0.00% |
| -3 | -1 | 3 | 19.47 | -7.5 | 122.71 | 1.019 | 1.019 | 0.01% |
| -3 | 1 | 3 | 19.48 | -2.5 | 95.98 | 1.018 | 1.019 | 0.06% |
| 1 | -3 | -3 | 19.47 | 1.5 | 241.26 | 1.019 | 1.019 | 0.01% |
| 3 | 1 | -3 | 19.46 | 15.5 | 302.51 | 1.019 | 1.019 | -0.04% |
| 0 | -4 | -2 | 19.97 | 3.0 | 218.18 | 0.993 | 0.993 | -0.04% |

**e**

| ***h*** | ***k*** | ***l*** | **2 (°)** | **  (°)** | ** ** | ***d_obs_* (Å)** | ***d_cal_* (Å)** | **Δ*d/d*** |
| --- | --- | --- | --- | --- | --- | --- | --- | --- |
| -1 | 1 | 2 | 10.91 | -15.1 | 83.99 | 1.812 | 1.812 | 0.03% |
| 1 | -1 | -2 | 10.91 | -3.8 | 263.94 | 1.812 | 1.812 | 0.03% |
| 1 | 2 | -1 | 10.90 | 11.2 | 344.86 | 1.814 | 1.812 | -0.06% |
| -2 | 0 | 2 | 12.59 | -1.3 | 110.02 | 1.571 | 1.570 | -0.08% |
| 2 | 0 | -2 | 12.60 | 11.8 | 290.09 | 1.570 | 1.570 | 0.00% |
| -2 | 2 | 2 | 15.44 | 0.3 | 74.81 | 1.282 | 1.282 | -0.05% |
| 2 | -2 | -2 | 15.45 | 15.7 | 254.68 | 1.281 | 1.282 | 0.01% |
| -2 | -1 | 3 | 16.69 | -19.5 | 124.45 | 1.187 | 1.187 | -0.02% |
| -2 | 1 | 3 | 16.69 | -13.0 | 93.10 | 1.187 | 1.187 | -0.02% |
| -1 | 3 | 2 | 16.71 | -13.6 | 55.03 | 1.185 | 1.187 | 0.09% |
| 2 | 1 | -3 | 16.70 | 0.9 | 304.24 | 1.186 | 1.187 | 0.04% |
| 0 | 4 | 0 | 17.85 | -6.6 | 19.05 | 1.110 | 1.110 | -0.03% |
| -3 | -1 | 3 | 19.47 | -7.7 | 124.00 | 1.019 | 1.019 | -0.01% |
| -3 | 1 | 3 | 19.47 | -3.5 | 96.96 | 1.019 | 1.019 | -0.01% |
| 3 | -1 | -3 | 19.48 | 15.8 | 277.02 | 1.018 | 1.019 | 0.04% |
| 3 | 1 | -3 | 19.47 | 15.5 | 303.83 | 1.019 | 1.019 | -0.01% |
| 0 | -4 | -2 | 19.99 | -0.8 | 220.23 | 0.992 | 0.993 | 0.03% |

**REFERENCES**

1. Uenver-Thiele L, Woodland A and Ballaran T *et al*. Phase relations of Fe-Mg spinels including new high-pressure post-spinel phases and implications for natural samples. *Am Mineral* 2017; **102**:2054-64.

2. Uenver-Thiele L, Woodland A and Ballaran T *et al*. Phase relations of MgFe_2_O_4_ at conditions of the deep upper mantle and transition zone. *Am Mineral* 2017; **102**:632-42.

3. Levy D, Diella V and Dapiaggi M *et al*. Equation of state, structural behaviour and phase diagram of synthetic MgFe_2_O_4_, as a function of pressure and temperature. *Phys Chem Miner* 2004; **31**:122-9.

4. Libowitzky E and Rossman GR. An IR absorption calibration for water in minerals. *Am Mineral* 1997; **82**:1111-5.
